# Synergistic Mechanisms of Surfactants and Monovalent Ions for Enhanced Oil Recovery Through Interfacial Properties and Microfluidic Study

**DOI:** 10.3390/gels12050435

**Published:** 2026-05-15

**Authors:** Xuchun Yang, Yafei Liu, Fen He, Chenlu Du, Jingdi Zheng, Desheng Zhou

**Affiliations:** 1 College of Petroleum Engineering, Xi’an Shiyou University, Xi’an 710065, China; 24212010117@stumail.xsyu.edu.cn (X.Y.); jianglu2540@163.com (C.D.); 25111010020@stumail.xsyu.edu.cn (J.Z.); dshzhou@xsyu.edu (D.Z.); 2 Anqing Branch, China Petroleum & Chemical Corporation, Anqing 246001, China; h3159852965@163.com

**Keywords:** surfactants, monovalent ions, microfluidics, interfacial properties, enhanced oil recovery

## Abstract

In oil and gas development, the oil displacement efficiency of single surfactants is inherently constrained. While synergistic interactions between salt ions and surfactants can enhance displacement performance by modulating interfacial properties and wettability, the underlying mechanisms remain insufficiently understood. This study systematically investigated the synergistic effects of two monovalent salts (NaCl, KCl) and four surfactants through macroscopic characterization of interfacial property and microfluidic displacement experiments using microfluidic device with dead-end structures. The results show that salt type and concentration significantly influence interfacial dynamics. The four selected surfactants exhibit gel-like behavior through molecular self-assembly in aqueous solutions, and their synergistic interaction with salt ions enhances oil displacement efficiency by modulating interfacial characteristics. High-salinity solutions reduce interfacial tension, with CTAB exhibiting a concentration-dependent decrease, while NP-10 behavior is governed by both surfactant and salt concentrations. The presence of Na^+^ generally resulted in lower IFT, improved interfacial viscoelasticity, and more favorable wettability alteration compared to K^+^. One-way analysis of variance confirmed that salt type is the main factor affecting recovery rate (*p* < 0.05). Notably, 0.2% CTAB+50,000 mg/L NaCl combination achieved the highest recovery rate owing to an optimal balance between interfacial adsorption, film viscoelasticity, and wettability alteration. This investigation elucidates the mechanisms driving surfactant–salt synergism and proposes an optimized surfactant and salt formulation to enhance oil recovery through tailored interfacial properties.

## 1. Introduction

As a strategic resource underpinning national energy security and economic development, efficient oil recovery is crucial for mitigating the growing imbalance between energy supply and demand. However, conventional primary and secondary oil recovery technologies typically achieve limited recovery rates of 30–40%, leaving a substantial volume of crude oil trapped in reservoirs. This necessitates the development of efficient enhanced oil recovery (EOR) technologies. Among EOR technologies, chemical flooding is considered as a key approach, involving the injection of chemical agents such as surfactants [1,2,3,4], polymers [5,6,7,8,9], and low-salinity water [10,11,12,13] to modify reservoir fluid properties and rock characteristics. It is important to note that surfactant flooding is distinct in its capacity to markedly reduce oil–water interfacial tension, modify rock wettability, and emulsify crude oil [14,15,16,17]. This demonstrates its significant value across a range of reservoir types by enhancing displacement efficiency. It has been demonstrated that monovalent salts (e.g., NaCl, KCl) have the capacity to enhance the interfacial stability of surfactants by regulating their interfacial dilatational rheological behavior [18]. In the study by Anto et al. [19], optical spectroscopy was employed to elucidate molecular interactions between crude oils and surfactant–salt systems. The results of this study revealed that anionic micelles selectively encapsulate two–four-ring polycyclic aromatic hydrocarbons (PAHs), while cationic/neutral systems capture two–six-ring compounds. This molecular-level insight facilitates the development of tailored surfactants for specific reservoirs. Li et al. [20] engineered EDAB, a thermal–viscoelastic surfactant, which exhibited superior performance to HPAM in incremental recovery, with an increase of 13.9% due to its enhanced heat/salt resistance and interfacial activity. Massarweh et al. [21] optimized Alfoterra surfactants for high-salinity carbonate reservoirs, thereby demonstrating 25.7~39.4% recovery gains over conventional flooding. These gains were achieved by leveraging salinity-enhanced interfacial activity and reduced cationic adsorption. Hama et al. [22] developed a peanut oil-derived anionic natural surfactant, which demonstrated excellent interfacial activity, wettability control ability and high salt emulsion stability. The recovery of oil was enhanced by up to 19.3% in experiments involving sandstone core flooding. The cationic high-branched PAMAM dendrimer surfactant G2-C12, synthesized by Abou-alfitooh et al. [23], has been shown to be capable of effectively reducing interfacial tension and reversing the wettability of sandstone. In a core flooding test, a recovery factor increase of 29.09% was observed.

Although surfactant flooding demonstrates efficacy across diverse reservoirs, single-system applications remain constrained by inherent limitations: in high-salinity reservoirs, salt sensitivity [24] readily weakens interfacial activity and solubility, bringing along challenges such as significant adsorption loss [25], high costs, and insufficient emulsification stability [26], which collectively restrict the application efficiency of surfactants.

In order to solve these problems, the combination of low-salinity water and surfactant flooding has gradually become a research hotspot and an important frontier in the field of EOR. Due to its outstanding synergistic advantages, it has received extensive attention from the academic community [27,28,29,30]. For example, Liu et al. [31] carried out targeted research through microscopic model experiments and analyzed the oil displacement effect of different salts. The results showed that low concentrations of NaCl and KCl can significantly improve oil recovery; the synergistic oil displacement efficiency of CaCl_2_ gradually increased with the increase in salinity. Kalam et al. [32] found that while low-salinity water alone has minimal impact on interfacial tension (IFT) reduction or wettability alteration, it significantly decreases the static adsorption of surfactants on carbonate rocks and maintains low dynamic adsorption during core displacement, laying the groundwork for low-cost, high-efficiency EOR processes. Molecular dynamics simulations further revealed that salt ions (e.g., Na^+^) can compress the electrical double layer around surfactant molecules, reducing their critical micelle concentration (CMC) and enhancing interfacial activity [33]. On this basis, Shakeel et al. [34] indicated that the combination of surfactant and salt solution can increase the recovery rate by 30~70%. Kedar et al. [35] further elucidated the synergistic interactions between salts and surfactants, revealing that Sodium Lauryl Ether Sulphate (SLES) exhibited the strongest synergy with salts, followed by MgCl_2_ > CaCl_2_ > NaCl; they attributed this to the salt-induced enhancement of surfactant distribution to the oil phase, enabling more efficient IFT reduction. Comparative studies reinforce these findings: Roldán–Carrillo et al. [29] reported that low-salinity water (LSW) with surfactant flooding increases recovery by 11.4 percentage points compared to 5.8 points for seawater alone, while Johannessen et al. [36] observed a 7.3 percentage point increase with low-salinity surfactant (LSS) versus 0.6 points for low-salinity water flooding. Meanwhile, studies have demonstrated that salt ions such as Na^+^ can significantly regulate the micellization behavior and interfacial distribution of surfactants [37,38].

Despite the confirmation of the synergistic oil displacement effect of salts and surfactants in the existing research, the interfacial interactions among surfactants, salts and crude oil remain incompletely understood. Consequently, the specific impact of such synergy on oil displacement efficiency remains ambiguous. In this study, the focus was on the investigation of two monovalent salts (NaCl, KCl) and four surfactants in relation to interfacial tension, dilatational modulus and wettability. A series of oil displacement tests were conducted employing dead-end pore structures for the efficient and rapid screening of the surfactant formula. In the cases of dead-end pores, the efficiency of oil recovery is the result of multiple mechanisms including the reduction in interfacial tension, emulsification, and wettability alteration. This approach also facilitates direct visual observation of crude oil displacement process at the micron scale, thereby enabling a systematic investigation of the microscopic mechanism governing the collaborative oil displacement of ions and surfactants.

## 2. Results and Discussion

### 2.1. Critical Micelle Concentration of the Surfactants

**Figure 1:**
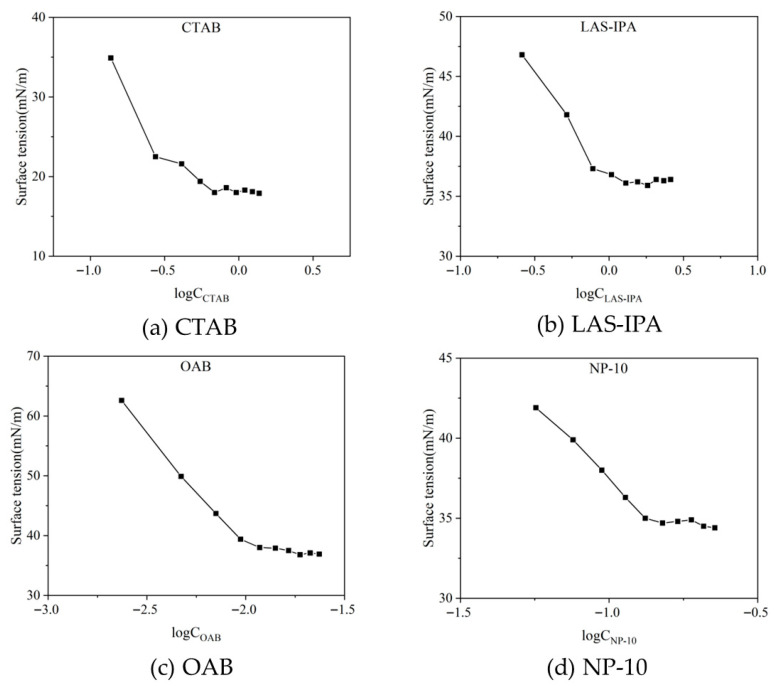
Surface tension versus solution concentration for four surfactants.

Measured surface tension for each surfactant was plotted versus the corresponding surfactant concentration. The inflection points on the curve represent the critical micelle concentration (CMC) values for each surfactant. As shown in Figure 1, the CMCs were determined as 0.686 mmol/L (0.025%), 0.778 mmol/L (0.027%), 0.012 mmol/L (0.00032%), and 0.132 mmol/L (0.0087%) for CTAB, LAS-IPA, OAB and NP-10, respectively. In this study, the applied surfactant concentrations were significantly higher than the minimum concentration required for micelle formation. Therefore, surfactant molecules could fully self-assemble into micelles, achieving a saturated and stable state of adsorption at the solution–oil interface.

### 2.2. Effect of Salts on the IFT and VE

**Figure 2:**
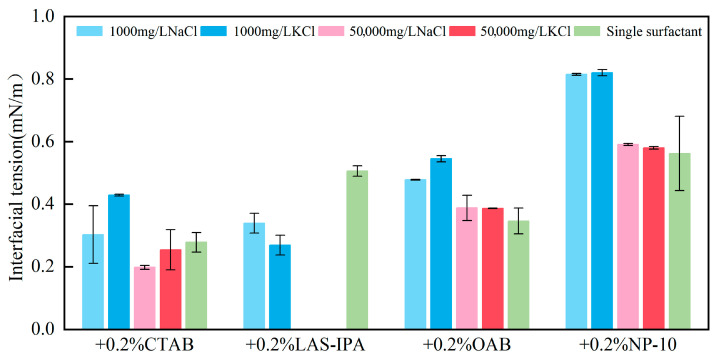
Measured interfacial tension between crude oil and different surfactant solutions.

**Figure 3:**
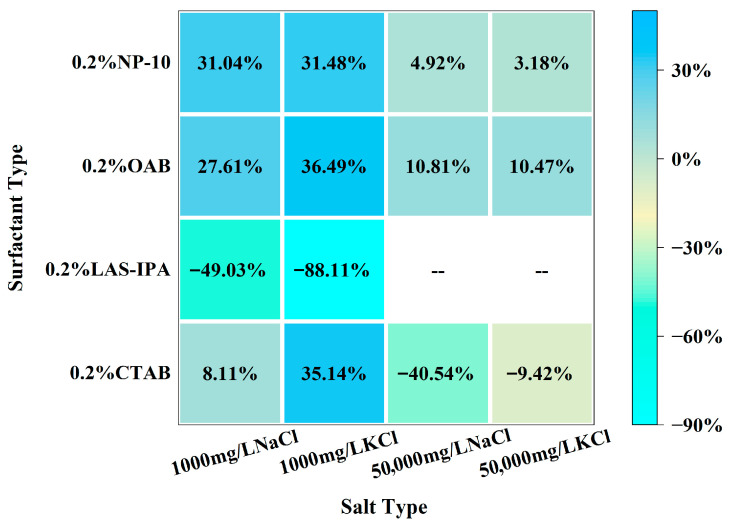
Heat map of the percentage change for interfacial tension between the composite solution and single surfactant.

As demonstrated in Figure 2, the interfacial tension of solutions comprising various surfactants and salts is exhibited. The combination of low-salinity brine and surfactant generally resulted in higher IFT compared with that of high-salinity brine and surfactant. In the majority of cases, the incorporation of NaCl brine resulted in a lower IFT compared to that of KCl brine. Specifically, for cationic surfactant CTAB, the addition of low-salinity brine could slightly increase the IFT, yet the addition of high-salinity brine resulted in lower IFT compared to the single surfactant. In the case of anionic surfactant LAS-IPA, the addition of low-salinity brine resulted in a lower IFT in comparison with the use of a single surfactant. In the case of CTAB, the addition of low-concentration salt has been shown to partially screen the electrostatic attraction between cationic headgroups of CTAB and interfacial sites. This has been demonstrated to result in a reduction in adsorption driving force and interfacial coverage, which in turn leads to increased IFT. In contrast, the application of low salinity in the context of LAS-IPA has been demonstrated to exert a positive influence on counterion binding to the anionic headgroups. This in turn has been shown to result in the suppression of electrostatic repulsion, thereby facilitating closer packing of the molecules. However, the high salt concentration increases the ionic strength of the solution, thereby triggering the salting-out effect. It has been demonstrated that the water solubility of LAS-IPA decreased sharply due to the competition between salt ions and LAS-IPA for water molecules, resulting in precipitation [39]. It has been shown that salinity exerts a comparable effect on the IFT for OAB and NP-10. In the case of zwitterionic surfactant OAB, the addition of low-concentration salts has been shown to result in the partial screening of internal charges. This leads to a less efficient packing of the molecules at the interface, consequently resulting in a slightly higher interfacial tension. The addition of low-concentration salts to nonionic surfactant NP-10 has been shown to influence the hydration of the ethoxylate group via a weak salting-out effect which has been demonstrated to reduce the interfacial adsorption efficiency, consequently resulting in higher IFT. In conditions of elevated salt concentration, there is an enhancement of electrostatic shielding and salting-out effects. These phenomena promote the adsorption of surfactants and interfacial packing, thereby restoring interfacial tension to levels that are comparable to those observed in single-surfactant systems. The comparison between the IFT of the composite solution and single surfactant was demonstrated in the heat map shown in Figure 3. These variations in IFT can be attributed to the differences in interfacial molecular arrangement and packing efficiency, which may also influence interfacial viscoelasticity and the formation of structured interfacial films with weak gel-like characteristics. In summary, the combinations of high-salinity brine with CTAB and low-salinity brine with LAS-IPA resulted in lower IFT.

**Figure 4:**
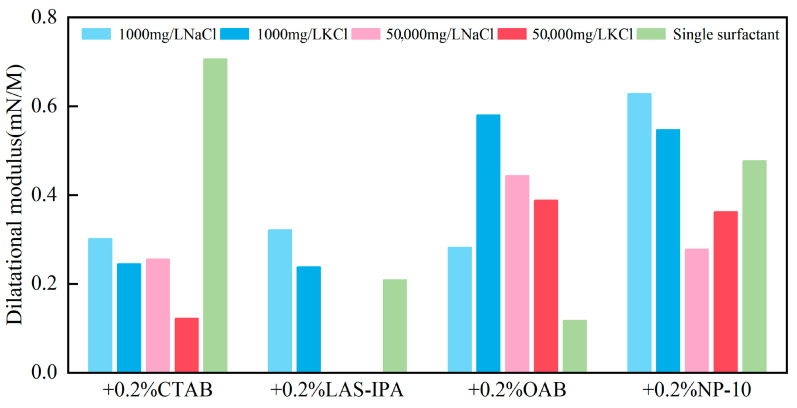
Calculated dilatational modulus between crude oil and different surfactant solutions.

The calculated dilatational modulus values for various composite solutions are shown in Figure 4.

For the cationic surfactant, the addition of brine solutions significantly reduced the dilatational modulus of the interface, indicating a weakening of interfacial structuring. For the anionic surfactant, brine solution has a slight impact on the dilatational modulus. Whereas for the zwitterionic surfactant, the addition of brine resulted in an evident increase in dilatational modulus, suggesting the formation or strengthening of a more robust gel-like structure at the interface. For nonionic surfactant, the combination of high-salinity brine and surfactant resulted in slightly lower dilatational modulus, indicating a less organized and weaker gel-like assembly, while the addition of low-salinity brine resulted in an increase in dilatational modulus, suggesting a moderate enhancement of the gel-like interfacial network.

**Figure 5:**
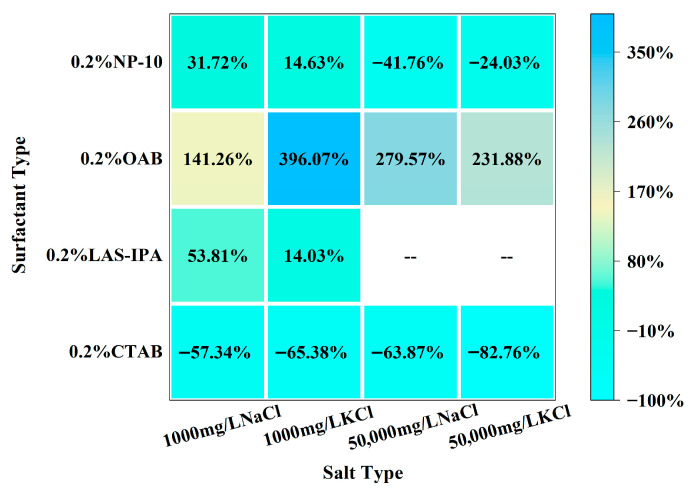
Heat map of the percentage change for dilatational modulus between the composite solution and single surfactant.

The comparison between the dilatational modulus of the composite solution and single surfactant was demonstrated in the heat map shown in Figure 5. For cationic surfactant CTAB, the addition of salt leads to charge screening and faster exchange kinetics. As a result, the interface could relax more quickly during the oscillation; therefore, dilatational modulus decreases. For LAS-IPA, the addition of low-concentration salt could enhance counterion binding to the anionic headgroups, resulting in suppressed electrostatic repulsion and closer packing of the molecules. Therefore, a denser interfacial layer could be formed, resulting in higher dilatational modulus. The zwitterionic surfactant possesses both positive and negative charges, and the addition of salt aids in screening intramolecular electrostatics and promoting a compact headgroup packing, resulting in an increase in dilatational modulus compared with the single surfactant. As for nonionic surfactant, mild salting-out at low salinity strengthens adsorption and elasticity, whereas higher salinity enhances bulk aggregation and faster exchange reduces the modulus.

### 2.3. Effect of Salts on the Wettability

**Figure 6:**
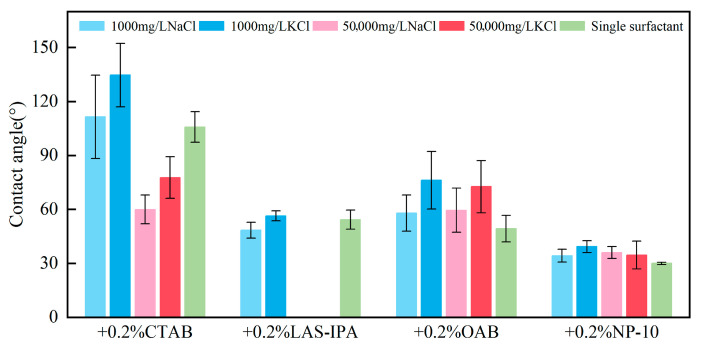
Measured contact angle between crude oil and different surfactant solutions.

**Figure 7:**
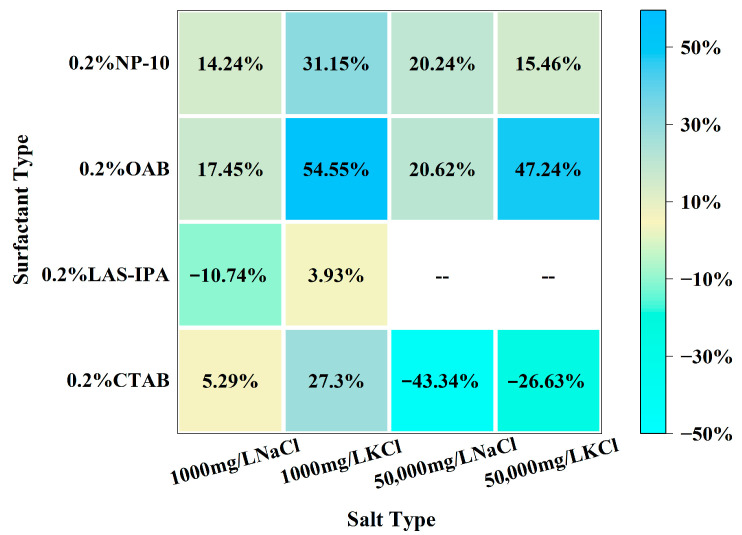
Heat map of the percentage change for contact angle between the composite solution and single surfactant.

As illustrated in Figure 6, the contact angle was measured for different composite solutions and crude oil samples. It was observed that the original contact angle for the surfactant-only system was comparatively low, with the exception of CTAB. Nevertheless, the incorporation of low-salinity brines into CTAB and OAB led to elevated contact angles, particularly in the presence of K^+^. Nonionic surfactant NP-10 demonstrated no discernible response to the addition of salts. Moreover, the presence of K^+^ generally resulted in a more oil-wet surface compared with that of Na^+^. In the case of cationic surfactant CTAB, the combination of lower-salinity brine and surfactant resulted in an oil–wet surface. The combination of higher-salinity brine and CTAB resulted in a surface that was more water-wet. In the case of anionic surfactant, the addition of low-salinity brine gave rise to an indistinct change in contact angle. In the case of zwitterionic surfactants, the incorporation of both low-salinity and high-salinity brines led to a higher contact angle. The incorporation of KCl-based solutions resulted in a comparatively higher increase in contact angle. The details of the change in contact angle are displayed in Figure 7. The investigation revealed that three combinations yielded lower contact angles, namely LAS-IPA with 1000 mg/L NaCl, CTAB with 50,000 mg/L NaCl, and CTAB with 50,000 mg/L KCl.

### 2.4. Oil Recovery with Composite Solutions

**Figure 8:**
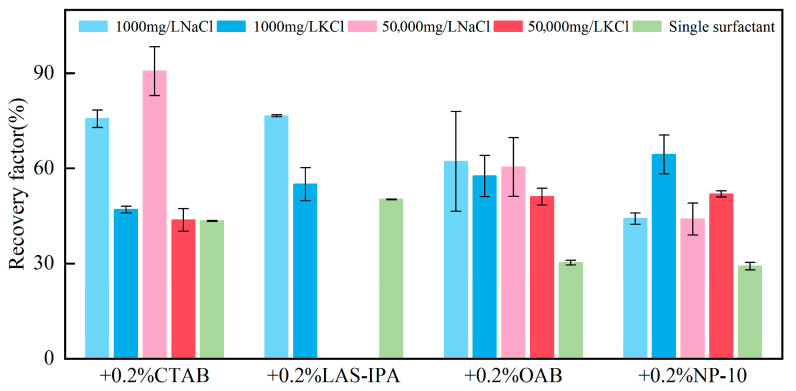
Oil recovery in the dead-end structures using different composite solutions.

**Figure 9:**
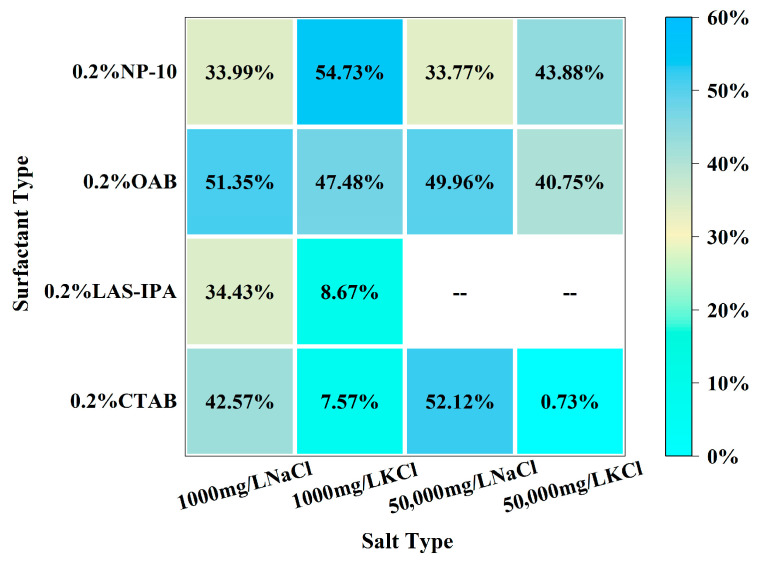
Heat map of the percentage change for oil recovery between the composite solution and single surfactant.

Microfluidic chips with triangular-shaped dead-end pores were employed to simulate the oil displacement process. The oil recovery rate using different combinations of surfactants and brines is plotted in Figure 8. In addition, oil displacement tests were conducted using single surfactants which served as the control group. The details of the change in oil recovery are displayed in Figure 9. In comparison with the control group, the incorporation of salt ions typically resulted in enhanced oil recovery. This observation suggests the presence of a synergistic effect between salt ions and surfactants. Oil recovery rate varied with the composition of the surfactant and salt solutions. Salt concentration has a higher impact on the oil recovery rate when the solution consists of CTAB. For other combinations of salts and surfactants, the impact of salt concentration is relatively minor. The highest oil recovery was achieved using the combination of CTAB and 50,000 mg/L NaCl. The superior performance of this system can be attributed to an optimal balance between electrostatic screening, interfacial packing, and film viscoelasticity at the molecular level. At high salinity, Na^+^ provides sufficient charge screening to reduce headgroup repulsion and promote dense interfacial packing, while its strong hydration prevents excessive screening that would otherwise weaken surfactant adsorption, resulting in maximal interfacial coverage and a uniform monolayer. The strong and stable adsorption of CTAB also facilitates wettability alteration toward more water-wet conditions. These synergistic effects collectively improve mobility control and oil mobilization, leading to the highest observed oil recovery. The characteristics of residual oil after different displacement tests are discussed in detail below.

#### 2.4.1. Cationic Surfactant CTAB with Salts

**Figure 10:**
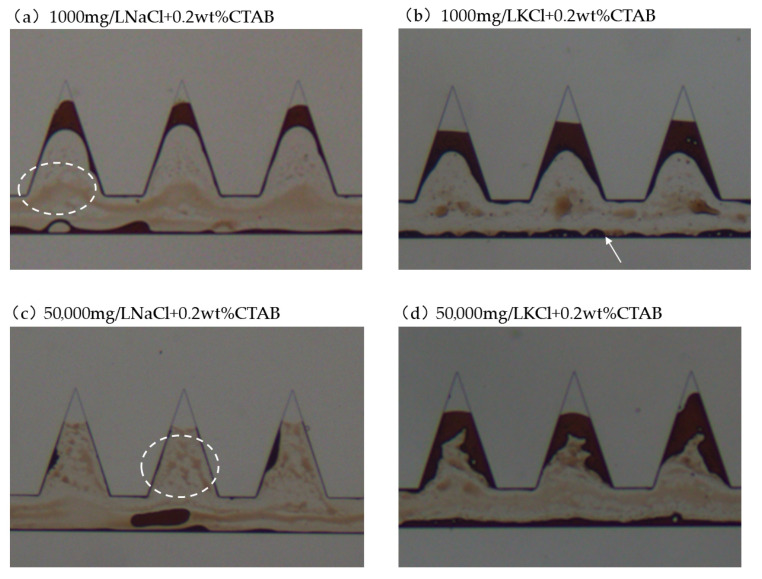
Residual oil characteristics in the dead-end pores when using CTAB with salts (dashed white circles indicate O/W emulsions and white arrow indicates W/O emulsions).

The combination of CTAB and salts generally resulted in lower IFT. Therefore, the oil phase was emulsified and dispersed in the aqueous phase. Additionally, a lighter brown color was frequently observed during the displacement process, indicating the plentiful formation of O/W emulsions indicated by the dashed white circles. Slight W/O emulsions were also observed indicated by the arrow. Particularly, 50,000 mg/L NaCl + CTAB resulted in the lowest IFT among the tested solutions. The emulsification process was more intense. Aqueous solution could penetrate into the apex, the dead-end pore. The residual oil characteristic after displacement tests using CTAB with different salts is shown in Figure 10. In Figure 10c, it was observed that oil slug was emulsified as the surfactant solution moved from the inlet to the outlet. It was evident that oil displacement efficiency was higher in the presence of Na^+^ than that of K^+^ when combined with cationic surfactant CTAB. The presence of Na^+^ resulted in lower IFT than that of K^+^. In addition, the dilatational modulus is higher in the presence of Na^+^. Owing to the stronger hydration of Na^+^ than K^+^, Na^+^ could provide moderate electrostatic screening while preserving the adsorption strength of surfactant at the interface, resulting in a more cohesive and viscoelastic interfacial film. This promotes the formation of stable, deformable emulsions with smaller droplet sizes. The superior oil recovery observed in the presence of Na^+^ arises from synergistic improvements in interfacial viscoelasticity and emulsion stability. In addition to the interfacial behaviors between surfactant solution and oil, the measured contact angle is also lower in the presence of Na^+^ than that of K^+^. Therefore, favored IFT, dilatational modulus and wettability resulted in better displacement performance in the presence of Na^+^.

#### 2.4.2. Anionic Surfactant LAS-IPA with Salts

**Figure 11:**
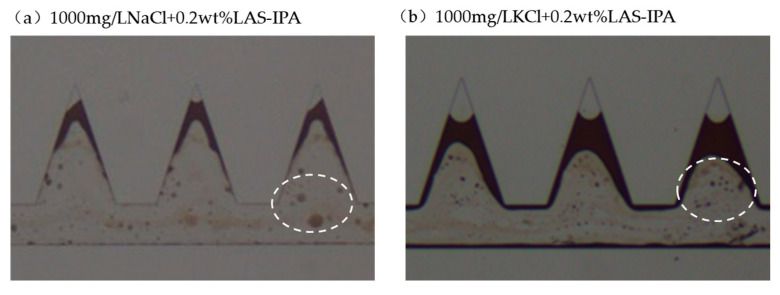
Residual oil characteristics in the dead-end pores when using LAS-IPA with salts (dashed white circles indicate O/W emulsion).

The residual oil characteristic after displacement tests using LAS-IPA with low-salinity salts is shown in Figure 11. The LAS-IPA and low-salinity brine system features low IFT, low dilatational modulus and low contact angle. Therefore, strong emulsification was observed. Compared with Figure 11a, relatively larger O/W emulsions were observed which could be attributed to its higher IFT indicated by dashed white circles. However, the combination of LAS-IPA and low-salinity brine demonstrated better capability in removing the oil film attached to the solid surface. This observation also aligns with the contact angle measurements. The solid surface carries negative charge when the pH is neutral. The addition of Na^+^ and K^+^ could partially neutralize the charge while forming a bridge for anionic surfactants. Overall, relatively higher oil recovery was achieved using low-salinity salt with anionic surfactant.

#### 2.4.3. Zwitterionic Surfactant OAB with Salts

**Figure 12:**
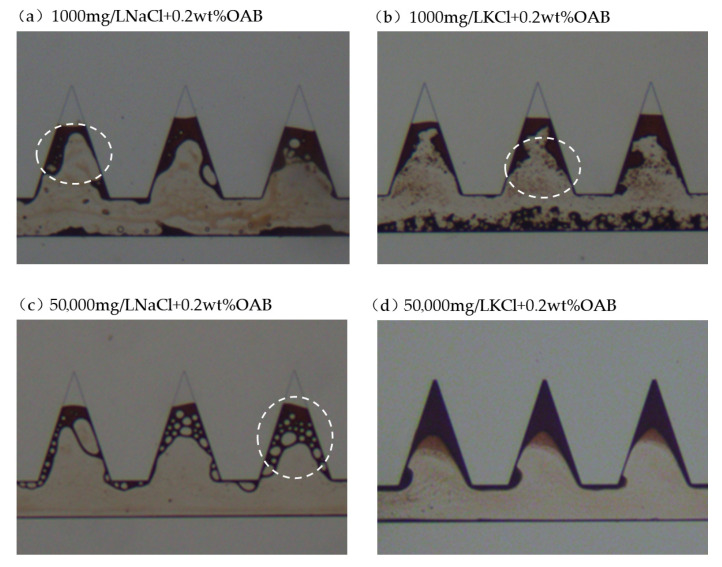
Residual oil characteristics in the dead-end pores when using OAB with salts (dashed white circles indicate W/O emulsions and O/W emulsions).

Figure 12 demonstrated the residual oil when using OAB with salts. The characteristics are different from the other experiments where W/O emulsions are frequently observed as shown in Figure 12a,c circled by white dashed lines. The zwitterionic surfactant resulted in relatively higher IFT and lower contact angle. Therefore, less oil film was attached to the solid surface and relatively larger emulsions were observed. The ultimate recovery was moderate after using OAB with salt solutions. The difference between adding high-salinity salt and low-salinity salt was not distinct, which could be attributed to the compatibility of zwitterionic surfactant with salts.

#### 2.4.4. Nonionic Surfactant NP-10 with Salts

**Figure 13:**
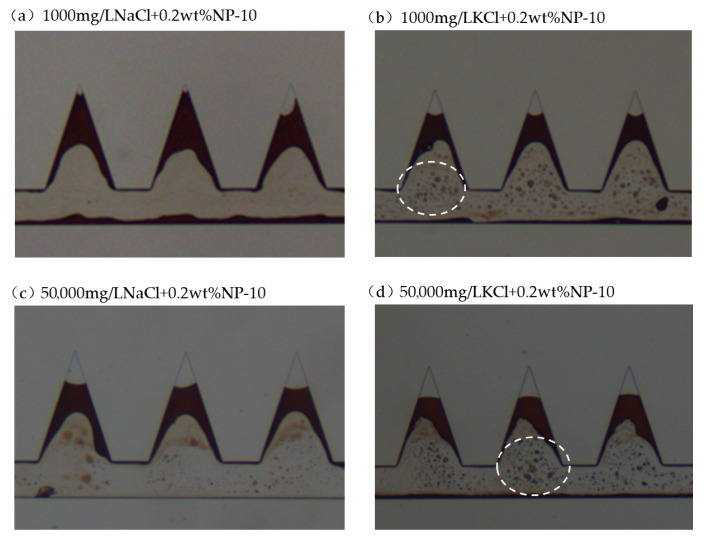
Residual oil characteristics in the dead-end pores when using NP-10 with salts (dashed white circles indicate O/W emulsion).

The residual oil characteristics after NP-10 and salt flooding are presented in Figure 13. O/W emulsions were observed and oil droplet size is generally larger compared to other cases due to relatively higher IFT. As shown in Figure 13a,c, emulsification between oil and water was not distinct whereas in Figure 13b,d, O/W emulsions were more frequently observed to be circled by white dashed lines. This observation aligns with the IFT measurement. The presence of K^+^ facilitated the emulsification process.

### 2.5. Statistical Analysis

Based on the oil recovery data presented above, analysis of variance (ANOVA) algorithm was employed to quantitatively evaluate the influence of three factors including surfactant type, salt type, and salt concentration on recovery efficiency. The algorithm assesses the significance of the impact of different levels of a single categorical factor on the recovery rate by calculating the F-value and the *p*-value. The F-value represents the ratio of the variance between groups to the variance within groups. Larger F-value indicates a more pronounced difference in recovery rates across different factor levels. The *p*-value is used to determine the statistical significance of the observed differences, with *p* < 0.05 as the threshold for statistical significance, and a smaller *p*-value indicating a stronger influence of the factor. The analytical results shown in Table 1 reveal that the F-value and *p*-value for salt type meet the *p* < 0.05 criterion, identifying it as the sole statistically significant factor. The factors are ranked by their F-values in descending order of influence which are salt type, surfactant type and salt concentration.

**Figure 14:**
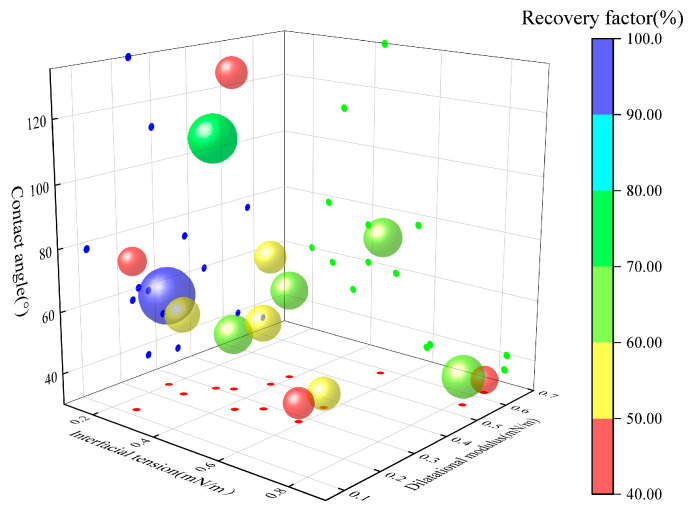
Relationship among interfacial tension, wettability, dilatational modulus and oil recovery.

The applicability of the dataset was first evaluated using the Shapiro–Wilk test for normality and Levene’s test for homogeneity of variance. Following validation, two-way ANOVA was conducted to assess the effects of surfactant type, salt type and concentration, and their interaction on interfacial tension, contact angle, and oil recovery. The main effects were interpreted based on the *p*-values of surfactant type and salt condition, while the interaction term was used to evaluate potential synergistic effects. The results in Table 2 show that all response variables satisfied the homogeneity of variance assumption, and that both main effects and their interaction were statistically significant, indicating a strong synergistic effect between surfactant and salt. Further analysis using Tukey’s HSD multiple comparison test identified the 0.2% CTAB + 50,000 mg/L NaCl system as the optimal formulation, exhibiting significantly enhanced oil recovery. Figure 14 depicts the relationship among measured IFT, dilatational modulus, contact angle and oil recovery rate in dead-end pores. As demonstrated in Figure 14, each bubble represents one set of experiment with recovery rate encoded by color. The system with a higher recovery rate typically exhibits characteristics of relatively low interfacial tension, moderate dilatational modulus and water-wet state. This finding is corroborated by the statistical analysis conclusions and intuitively demonstrates the pivotal role of interfacial characteristic optimization in enhancing oil recovery rate.

## 3. Conclusions

This study systematically investigated the interfacial behavior and enhanced oil recovery performance of four surfactants (CTAB, LAS-IPA, OAB, NP-10) in the presence of two monovalent salts (NaCl, KCl) under varying salinity conditions. The results demonstrate that increasing salinity generally reduces interfacial tension across most systems, although the extent of reduction depends strongly on surfactant type. CTAB exhibits pronounced sensitivity to salinity, while LAS-IPA achieves substantial interfacial tension reduction but suffers from precipitation at elevated salt concentrations. For dilatational modulus, OAB shows a significant increase up to 396.07% at 1000 mg/L KCl, indicating enhanced interfacial structuring and elasticity with gel-like characteristics. In contrast, CTAB displays a decrease in dilatational modulus with increasing salinity, suggesting a weakening of interfacial film strength. Wettability measurements reveal that NP-10 produces the lowest contact angle and remains largely insensitive to salinity variations, whereas CTAB shows strong salinity-dependent wettability alteration, with higher salt concentrations promoting more water-wet conditions. OAB and LAS-IPA exhibit comparatively stable wettability behavior in response to salt addition. Oil displacement experiments confirm that the incorporation of salts improves recovery efficiency for all surfactant systems relative to single-surfactant flooding. Solutions containing Na^+^ achieve an average recovery factor of 64.81%, outperforming those with K^+^, highlighting the superior synergistic effect of sodium ions. A total of 0.2% CTAB combined with 50,000 mg/L NaCl yields a maximum recovery of 90.72%, which could be attributed to enhanced emulsification, improved fluid mobility, and reduced oil film attachment. The presence of Na^+^ ions favors the formation of oil-in-water emulsions with greater oil-carrying capacity, thereby facilitating more effective displacement. Overall, this study provides mechanistic insights into the role of monovalent salts in modulating surfactant performance and offers a practical basis for designing synergistic surfactant–salt systems for EOR applications.

## 4. Materials and Methods

### 4.1. Materials

**Figure 15:**
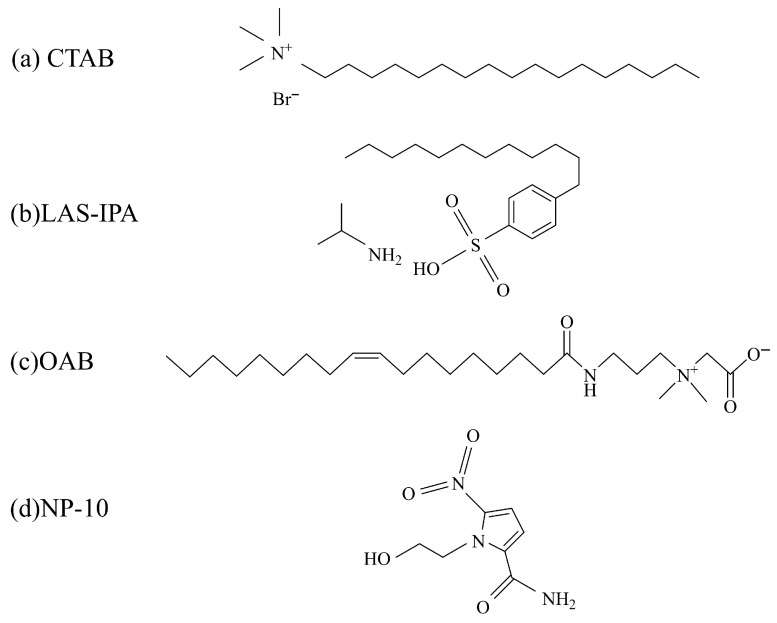
Chemical structure of the surfactants used.

The information of reagent used in the experiment is shown in Table 3. Four different surfactants were employed including cetyltrimethylammonium bromide (CTAB), isopropylammonium dodecylbenzenesulfonate (LAS-IPA), oleoylamidopropyl betaine (OAB), and nonylphenol polyoxyethylene ether (NP-10). Their chemical structures are shown in Figure 15. Formation water solution with 50,000 mg/L salinity (80% NaCl, 3% KCl, 7% MgCl_2_, 10% CaCl_2_) was prepared. The composition of the crude oil used in the experiment is shown in Table 4. The four surfactants were combined with the two inorganic salts (NaCl and KCl with 1000 and 50,000 mg/L salinity) to produce the composite solutions shown in Table 5. Surfactant concentration was determined according to the range of surfactant concentration applied in the field. Na^+^, K^+^ and Cl^−^ were selected considering that they are the most common monovalent ions observed in the formation water. Salinity of 1000 mg/L is the typical concentration of low-salinity water flooding, and 50,000 mg/L salinity is consistent with the total salinity of the formation water. The pH of tested solutions ranged from 5 to 6. It was noted that 50,000 mg/L NaCl with 0.2 wt% LAS-IPA and 50,000 mg/L KCl with 0.2 wt% LAS-IPA exhibited turbidity or precipitation, rendering accurate measurement of interfacial properties and oil displacement efficiency impossible. Therefore, only clear solutions were selected for subsequent experiments. The experiments were conducted at room temperature and atmospheric pressure.

### 4.2. Methods

#### 4.2.1. Determination of the Critical Micelle Concentration

The surface tension method was used to determine the critical micelle concentration (CMC) of the surfactants.

#### 4.2.2. Determination of the Interfacial Tension and Dilatational Modulus

**Figure 16:**
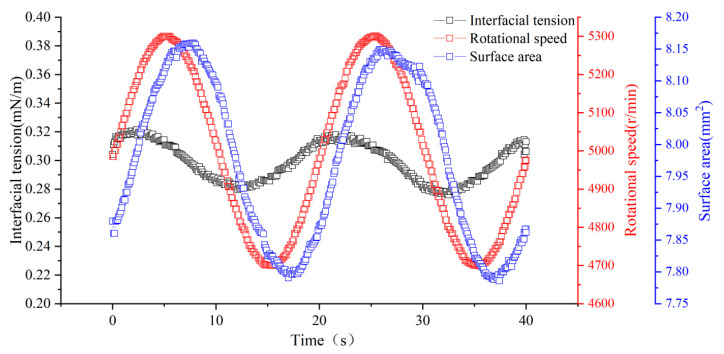
Schematic diagram of the sinusoidal relationship curve between time, interfacial tension, surface area, and rotational speed.

Interfacial tension was measured using a rotating drop tension meter (CNGTX 701, Beijing Shengwei Technology Co., Ltd., Beijing, China). The measured solution was injected into a glass tube, and then oil droplets were introduced into the solution using a micro-syringe. Interfacial tension between different solutions and crude oil was measured at 30 °C and a rotation speed of 5000 rpm. An equilibration period of 40 min was applied for all systems to ensure sufficient interfacial adsorption prior to measurement. Interfacial tension values were recorded after stabilization, thereby minimizing the influence of transient, non-equilibrium effects. In addition, each measurement was repeated multiple times, and the reported error bars represent standard deviations. A sinusoidal relationship curve as shown in Figure 16 was obtained. The interfacial tension of the oil droplet was calculated using the Young–Laplace equation, revealing the correlation between surface area, time, and interfacial tension. Dilatational modulus (E*) was calculated using Equation (1) [40].

(1) $$E*=\frac{∆\gamma }{∆A/A_{0}}$$

where ∆γ is the amplitude of interfacial tension, mN/m; ∆A is the amplitude of surface area, mm^2^; A_0_ is the average surface area, mm^2^.

#### 4.2.3. Determination of the Contact Angle

Contact Angle was measured using a Video Optical Contact Angle Measuring Instrument (KRÜSS GmbH, Hamburg, Germany). The captive bubble method was employed to measure the contact angles between crude oil and the composite solution [41]. Glass slides were aged in crude oil in a 30 °C water bath for 24 h. Afterwards, the glass slides were rinsed with kerosene. The aged slide was secured in a distilled water container. A droplet of oil was injected beneath the slide using a curved syringe and the initial contact angle was measured. The slides were then placed in the tested solution and aged at 30 °C for 24 h. The above procedure was repeated to measure the contact angle between crude oil and the tested composite solution. The measurements were repeated multiple times. The error bars in the figure represent the standard deviations from multiple measurements.

#### 4.2.4. Microscale Oil Displacement Experiment

The fabrication of the microfluidic chip follows the standard photolithography and soft lithography methods. First, a silicon wafer featuring the triangular-shaped dead-end structure was fabricated via photolithography to serve as a master mold. The mold was then securely fixed, and uncured polydimethylsiloxane (PDMS) was poured over it. Afterwards, the assembly was cured in the oven at 65 °C for 6 h. The solidified PDMS was then carefully peeled off from the silicon mold. Holes were then punched into the PDMS to provide inlet and outlet. Finally, the PDMS and a thoroughly cleaned glass slide were treated in a plasma cleaner to achieve permanent bonding, thereby yielding a complete microfluidic chip. Prior to each oil displacement test, a fresh microfluidic chip was fabricated to ensure a consistent initial condition.

**Figure 17:**
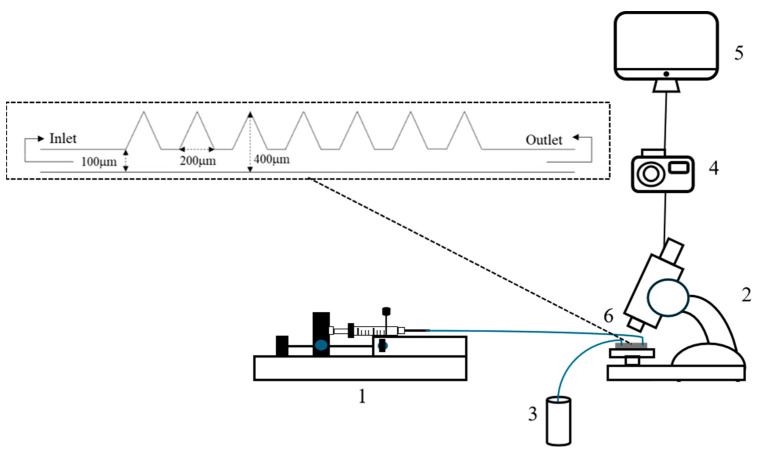
Schematic of oil displacement experiment setup (1: pump and syringe; 2: microscope; 3: waste liquid collection; 4: high-speed camera; 5: computer; 6: microfluidic chip).

The oil displacement experiment device mainly consists of an inverted microscope, a high-speed camera, a computer, and an injection pump which is shown in Figure 17. The outlet end of the syringe is connected to the inlet port of the microfluidic chip via tubings. The initial water saturation was established by injecting formation water at a rate of 5 μL/min until the saturation is stable. The initial oil saturation was established by injecting crude oil at a rate of 2 μL/min until the saturation is constant. Then, the composite solution was injected at a rate of 2 μL/min. Photographs were taken at 1 min, 2 min, 3 min, 4 min, 5 min, 8 min, 11 min, 16 min, 21 min, 31 min, and 41 min of displacement, respectively. The experiment procedures were repeated using different solutions. Finally, ImageJ software (NIH, 1.54r) was used to binarize the images captured by the high-speed camera and the recovery rate was calculated using Equation (2).

(2) $$\eta =(1-\frac{S_{or}}{S_{oi}})$$

where η is the recovery rate, %; $S_{or}$ is residual oil saturation; $S_{oi}$ is the initial oil saturation.

## Figures and Tables

**Table 1:** Statistical analysis of the dominant factor.

Influencing Factor	F-Value	*p*-Value	Significance
Salt Type	8.30	0.0064	Significant
Surfactant Type	2.04	0.1245	Not Significant
Salt Concentration	0.34	0.5613	Not Significant

**Table 2:** Statistical analysis of the applicability of the dataset.

Evaluation Index	Interfacial Tension	Contact Angle	Oil Recovery
Normality test *p*-value	0.0061	0.0003	0.0041
Homogeneity test *p*-value	0.0159	0.3370	0.1154
Surfactant main effect *p*-value	<0.001	<0.001	<0.001
Salt condition main effect *p*-value	<0.001	0.0226	<0.001
Interaction *p*-value	0.0009	<0.001	<0.001

**Table 3:** Details of the materials used in the experiment.

Reagent	Producer
NaCl	Shanghai Aladdin Biochemical Technology Co., Ltd. (Shanghai, China)
CaCl_2_	Tianjin Tianli Chemical Reagent Co., Ltd. (Tianjin, China)
KCl	Tianjin Tianli Chemical Reagent Co., Ltd. (Tianjin, China)
MgCl_2_·6H_2_O	Tianjin Tianli Chemical Reagent Co., Ltd. (Tianjin, China)
CTAB	Chemical Reagent Co., Ltd. (Shanghai, China)
LAS-IPA	Shandong Yousuo Chemical Technology Co., Ltd. (Zibo, China)
OAB	Shandong Yousuo Chemical Technology Co., Ltd. (Zibo, China)
NP-10	Shanghai McLean Biochemical Technology Co., Ltd. (Shanghai, China)

**Table 4:** Crude oil composition.

Asphaltene/%	Resin/%	Aromatics/%	Saturates/%
2.09	22.78	4.27	70.86

**Table 5:** Detailed composition of the tested solutions.

No.	NaCl and Surfactant	No.	KCl and Surfactant
1	1000 mg/LNaCl + 0.2 wt%CTAB	9	1000 mg/LKCl + 0.2 wt%CTAB
2	1000 mg/LNaCl + 0.2 wt%LAS-IPA	10	1000 mg/LKCl + 0.2 wt%LAS-IPA
3	1000 mg/LNaCl + 0.2 wt%OAB	11	1000 mg/LKCl + 0.2 wt%OAB
4	1000 mg/LNaCl + 0.2 wt%NP-10	12	1000 mg/LKCl + 0.2 wt%NP-10
5	50,000 mg/LNaCl + 0.2 wt%CTAB	13	50,000 mg/LKCl + 0.2 wt%CTAB
6	50,000 mg/LNaCl + 0.2 wt%LAS-IPA	14	50,000 mg/LKCl + 0.2 wt%LAS-IPA
7	50,000 mg/LNaCl + 0.2 wt%OAB	15	50,000 mg/LKCl + 0.2 wt%OAB
8	50,000 mg/LNaCl + 0.2 wt%NP-10	16	50,000 mg/LKCl + 0.2 wt%NP-10

## Data Availability

The authors confirm that the data are provided within the article.
